# Was it a pain or a sound? Across-species variability in sensory sensitivity

**DOI:** 10.1097/j.pain.0000000000000316

**Published:** 2015-08-06

**Authors:** Li Hu, Xiaolei L. Xia, Weiwei W. Peng, Wenxin X. Su, Fei Luo, Hong Yuan, Antao T. Chen, Meng Liang, Giandomenico Iannetti

**Affiliations:** a Key Laboratory of Cognition and Personality (Ministry of Education) and Faculty of Psychology, Southwest University, Chongqing, China; b Department of Neuroscience, Physiology and Pharmacology, University College London, London, United Kingdom; c Key Laboratory of Mental Health, Institute of Psychology, Chinese Academy of Sciences, Beijing, China

**Keywords:** Sensory sensitivity, Aδ-fibres, C-fibres, Electroencephalogram (EEG), Rats, Humans

## Abstract

Natural selection has shaped the physiological properties of sensory systems across species, yielding large variations in their sensitivity. Here, we used laser stimulation of skin nociceptors, a widely used technique to investigate pain in rats and humans, to provide a vivid example of how ignoring these variations can lead to serious misconceptions in sensory neuroscience. In 6 experiments, we characterized and compared the physiological properties of the electrocortical responses elicited by laser stimulation in rats and humans. We recorded the electroencephalogram from the surface of the brain in freely moving rats and from the scalp in healthy humans. Laser stimuli elicited 2 temporally distinct responses, traditionally interpreted as reflecting the concomitant activation of different populations of nociceptors with different conduction velocities: small-myelinated Aδ-fibres and unmyelinated C-fibres. Our results show that this interpretation is valid in humans, but not in rats. Indeed, the early response recorded in rats does not reflect the activation of the somatosensory system, but of the auditory system by laser-generated ultrasounds. These results have wide implications: retrospectively, as they prompt for a reconsideration of a large number of previous interpretations of electrocortical rat recordings in basic, preclinical, and pharmacological research, and prospectively, as they will allow recording truly pain-related cortical responses in rats.

## Abstract 2

Supplemental Digital Content is Available in the Text.

Valid translation of experimental results from animals to humans critically relies on a careful consideration of differences in sensory sensitivity across species.

## 1. Introduction

The sensitivity of sensory systems varies greatly across species. For example, the human auditory system is sensitive to sonic frequencies (20-20,000 Hz), whereas some other animals can detect infrasounds or ultrasounds.^39^ Rats use ultrasonic calls for social communications, and ultrasound sensitivity is a hallmark of other non-human species, including bats, dolphins, and dogs.^41^

Correct translation of experimental results from animals to humans relies on a careful consideration of the different sensitivity of sensory systems across species.^58^ Overlooking such differences could lead to incorrect interpretations of experimental data and generate important misconceptions. Here, we provide a vivid example of this issue in sensory neuroscience and translational medicine.

Laser stimulators currently represent the most accurate tool for experimental studies of pain,^6^ as they activate Aδ- and C-nociceptors selectively and elicit sensations of “pure” pain without touch.^2,4,14,15^ For these reasons, laser stimulation is considered the gold standard to investigate pain psychophysically and electrophysiologically, and it has been used in hundreds of human and rat studies.^7,12,13,24,25,28,34,45,46,48,52,55,57^ Because of the different conduction velocity of Aδ (∼15 m/s) and C (∼1 m/s) afferents, laser pulses elicit a double sensation: an initial Aδ-related pricking pain is followed by a C-related burning pain.^30^ In humans, temporally distinct transient responses in the electroencephalogram (EEG) (event-related potentials, ERPs) correspond to these 2 sensations.^17^ Consistent with human studies, it is commonly reported that laser-evoked ERPs in freely moving rats also show different components at latencies compatible with the conduction velocity of Aδ-fibres (“Aδ-ERPs”) and C-fibres (“C-ERPs”).^21,28,46,52,53,57,62^

However, the interpretation of the responses recorded in those studies ignores 2 important facts. First, when delivered on the skin of humans and animals, laser pulses not only induce a steep temperature increase that activates nociceptors^54^ but also generate a broadband ultrasound through a thermo-elastic mechanism, ie, the sudden thermal expansion due to the heating of the surface of the irradiated material.^31,49,61^ This laser-generated ultrasound occurs in a frequency band (∼50 kHz) that is heard by rats,^41^ but not by humans. Second, as revealed by microneurographical recordings from single peripheral axons,^9,33^ conduction velocity assessment of peripheral afferents,^23^ and nocifensive behaviors,^10^ Aδ-fibres are virtually never activated by heat in rats. Thus, laser-induced heat is effectively detected by Aδ-fibres in humans, but not in rats.

Considering these 2 striking differences in the sensory sensitivity of both auditory and nociceptive systems between rats and humans, it is surprising that plenty of studies keep ascribing the early part of laser-evoked rat brain responses to the activation of pain-related cutaneous Aδ-nociceptors (“Aδ-ERPs”).^21,28,46,51–53,57,62^ Instead, a physiologically more viable hypothesis is that such “Aδ-ERPs” reflect the activation of the auditory system by laser-generated ultrasounds. Here, in 6 different experiments, we tested this hypothesis, by comprehensively characterizing and comparing the physiological properties of the electrocortical responses elicited by laser stimulation in rats and humans. We recorded the EEG from the surface of the brain (in 29 freely moving rats) and from the scalp (in 15 healthy humans).

## 2. Methods

### 2.1. Animal experiments

#### 2.1.1. Animal preparation and surgical procedures

A total of 29 adult male Sprague–Dawley rats weighing 300 to 400 g were used. The rats were housed in cages under temperature-controlled and humidity-controlled conditions. All rats received food and water ad libitum and were kept in a 12-hour day–night cycle (light on from 08:00-20:00). All surgical and experimental procedures were approved by the local ethics committee of Southwest University (Chongqing, China).

Before the surgery, rats were anesthetized with sodium pentobarbital (50 mg/kg, intraperitoneal injection: i.p.). Supplementary doses (12.5 mg/kg, i.p.) of sodium pentobarbital were given to maintain appropriate anesthetic depth during surgery, when necessary. As described in previous studies,^46,51,52^ during anesthesia, the rat head was fixed using a stereotaxic apparatus, and after the dorsal aspect of the scalp was shaved, the skull was exposed by a midline incision. Fourteen holes were drilled on the skull, at locations based on the stereotaxic reference system.^51,52^ Stainless steel screws (outside diameter = 1 mm) were inserted into the holes without penetrating the underlying dura. Twelve screws acted as active electrodes, placed according to the position of bregma (Supplementary Fig. 1, available online as Supplemental Digital Content at http://links.lww.com/PAIN/A143). The reference and ground electrodes were, respectively, placed 2 and 4 mm caudally to the lambda, on the midline. The wires coming from each electrode were held together with a connector module fixed on the scalp with dental cement. To prevent postsurgical infections, rats were injected with penicillin (60,000 U, i.p.). After the surgery, rats were kept in individual cages for at least 7 days before the EEG experiments.

#### 2.1.2. Experimental protocol

Radiant-heat stimuli were generated by an infrared neodymium yttrium aluminum perovskite (Nd:YAP) laser with a wavelength of 1.34 μm (Electronical Engineering, Italy). Laser pulses were delivered on a predefined skin area, and activated directly nociceptive terminals in the most superficial layers.^2,20^ The stimulated areas were defined depending on the objective of each experiment, as detailed below. The laser beam was transmitted through an optic fibre, and its diameter was set at ∼4 mm (13 mm^2^) by focussing lenses. A He-Ne laser pointed to the stimulated area. The laser pulse duration was 4 milliseconds, and the interstimulus interval was never shorter than 30 seconds. To avoid nociceptor fatigue or sensitization, the target of the laser beam was displaced after each stimulus.

During EEG data collection, rats were placed into a plastic cage (30 × 30 × 40 cm^3^), whose bottom side had regular series of holes through which the animal could be stimulated. The diameter of each hole was 5 mm. The ceiling of the cage had a single hole (diameter 15 cm) through which the EEG cables were passed (Supplementary Fig. 1, available online as Supplemental Digital Content at http://links.lww.com/PAIN/A143). Before the EEG experiments, rats were placed for at least 4 slots of 1 hour each in the same plastic cage subsequently used for the EEG experiment. This procedure allowed rats to become familiar with the recording environment.

Both in the prerecording and recording sessions, rats could freely move in the cage. Laser pulses were delivered through the holes in the bottom side of the cage when the rats were spontaneously still. Data for each of the first 4 experiments were collected from 16 rats. Data for Experiment 5 were collected from 13 different rats. Details of each experiment follow.

##### 2.1.2.1. Experiment 1

We assessed the effect of stimulus intensity and stimulation site on laser-evoked ERPs and estimated the conduction velocity of afferent fibres mediating the EEG responses. We delivered 15 laser pulses at each of 5 laser energies (E1-E5, 1.5 J-3.5 J, in steps of 0.5 J) and at each of 2 stimulation sites (tail base and tail tip, in alternated trials), for a total of 150 pulses. The order of stimulus intensities was pseudorandomized.

##### 2.1.2.2. Experiment 2

We assessed the effect of ongoing white noise on the ERPs elicited by laser pulses delivered at different sites. The experiment consisted of 2 blocks, one with ongoing white noise (90 dB, the same intensity was used in Experiments 3 and 4), the other without white noise. In each block, we delivered 15 laser pulses (3.5 J) at each of 2 stimulation sites (tail base and tail tip, in alternated trials). The order of blocks was balanced across rats. The white noise was generated using GoldWave Digital Audio Editor (GoldWave Inc.) and had a bandwidth of 0 to 22,050 Hz.

##### 2.1.2.3. Experiment 3

We investigated whether laser stimuli not delivered on the skin but in the environment surrounding the animal were still able to evoke a brain response. The experiment consisted of 2 blocks, one with ongoing white noise and the other without ongoing white noise. In each block, we delivered 30 laser pulses (3.5 J) on the cage, at ∼5 to 10 cm from the rat. The order of blocks was balanced across rats.

##### 2.1.2.4. Experiment 4

We tested the possible influence of stimulated skin type on laser-evoked ERPs. The experiment consisted of 2 blocks, one with ongoing white noise and the other without ongoing white noise. In each block, we delivered 15 laser pulses (3.5 J) at each of 2 stimulation sites (glabrous skin of the left forepaw and hairy skin of the left foreleg, in alternated trials). The order of blocks was balanced across rats. To ensure stimulation of skin nociceptors, the left foreleg was shaved 2 days before the experiment.

##### 2.1.2.5. Experiment 5

Because Experiments 1 to 4 demonstrated that “Aδ-ERPs” reflected the activation of the auditory system by laser-generated ultrasounds, Experiment 5 was performed during ongoing white noise (60 dB) to selectively characterize the ERPs related to the activation of the nociceptive system. We delivered 10 laser pulses at each of 5 stimulus intensities (E1′-E5′, 1-4 J in steps of 0.75 J) and at each of 4 stimulation sites (left forepaw, right forepaw, left hindpaw, and right hindpaw), for a total of 200 pulses. The order of stimulus intensities and stimulation sites was pseudorandomized. The rat behavior was video-recorded throughout the experiment, and behavioral scores were assigned based on the animal movements after each laser stimulus, according to previously defined criteria.^10,11^ Specifically, stimulus-evoked nocifensive behaviors were classified into 5 types as follows: no movement (score = 0), head turning (including shaking or elevating the head; score = 1), flinching (ie, a small abrupt body jerking movement; score = 2), withdrawal (ie, paw retraction from the laser stimulus; score = 3), and licking and whole-body movement (score = 4).

#### 2.1.3. Electroencephalogram recording and data analysis

Cortical activity was recorded from 12 active electrodes, amplified, and digitized using a sampling rate of 1000 Hz (Brain Products). Electroencephalogram data were preprocessed using EEGLAB,^8^ an open source toolbox running in the MATLAB environment. Continuous EEG data were band-pass filtered between 1 and 30 Hz. Electroencephalogram epochs were extracted using a window analysis time of 1500 milliseconds (500 milliseconds before stimulus and 1000 milliseconds after stimulus) and baseline corrected using the prestimulus interval. Trials contaminated by gross artifacts were manually rejected for the following analysis.

For each rat and experimental condition, average ERP waveforms were computed, time-locked to the onset of the laser stimulus. Single-rat average waveforms were subsequently averaged to obtain group-level average waveforms. Group-level scalp topographies were computed by spline interpolation. The boundary of the scalp topography was determined based on stereotaxic coordinates.^43^

In Experiments 1 to 4, peak latencies and amplitudes of P1, N1, and N2 waves were measured in each rat and condition, from the ERP waveforms obtained by pooling data recorded from the 4 central electrodes (FL2, FR2, PL1, and PR1, ie, the electrodes at which these waves showed a maximal peak amplitude). In Experiment 5, we demonstrated the spatial correspondence between the scalp distribution of the early part of the C-ERPs elicited by forepaw stimulation (∼120 milliseconds) and hindpaw stimulation (∼200 milliseconds) and the anatomical location of the primary somatosensory cortex (S1). The locations of the forelimb and hindlimb S1 were determined using the 3D Paxinos and Watson atlas of the rat brain^16,43^ and displayed on the 3D brain mask surface from the Waxholm Space atlas of the rat brain.^1,42^

### 2.2. Human experiment

#### 2.2.1. Participants

Fifteen healthy volunteers (7 females) aged 22.4 ± 1.8 years (mean ± SD) participated in the study. All volunteers gave their written informed consent and were paid for their participation. The experimental procedures were approved by the local ethics committee of Southwest University (Chongqing, China).

#### 2.2.2. Experimental protocol

Radiant-heat stimuli used in the human experiment were identical to those used in the animal experiments, except for the laser beam diameter, which was set at ∼7 mm (38 mm^2^). Laser pulses were delivered to the dorsum of the left hand and of the left foot. After each stimulus, the target of the laser beam was shifted by at least 1 cm in a random direction, to avoid increases in baseline skin temperature and nociceptor fatigue or sensitization. Participants were asked to report the subjective intensity of the pain perception elicited by each laser stimulus, using a numerical rating scale ranging from 0 (“no pain”) to 10 (“pain as bad as it could be”), with 4 denoting pinprick pain threshold.^17^

##### 2.2.2.1. Experiment 6

We assessed the effect of ongoing noise on the human ERPs elicited by laser pulses delivered on the dorsum of the hand and of the foot. The experiment consisted of 4 separate blocks. The order of blocks was balanced across the 15 participants. In each block, laser pulses were delivered to either the hand dorsum or the foot dorsum, either with or without ongoing white noise. The intensity of white noise (90 dB) was the same used in the animal recordings (Experiments 1-5). Since in previous studies we observed that a graded stimulation is optimal to detect both Aδ-ERPs and C-ERPs,^17^ in each block we delivered 10 laser pulses at each of 4 laser energies (hand blocks: E1, 2.47 ± 0.23 J; E2, 2.97 ± 0.23 J; E3, 3.47 ± 0.23 J; and E4, 3.97 ± 0.23 J and foot blocks: E1, 2.85 ± 0.21 J; E2, 3.35 ± 0.21 J; E3, 3.85 ± 0.21 J; and E4, 4.35 ± 0.21 J), for a total of 40 pulses. The energies of stimulation were determined for each individual in a preliminary session, as follows: the highest energy (E4) corresponded to a rating of 8 out of 10 and the lower energies (E1-E3) were defined by progressively subtracting 0.5 J. The order of stimulus energies was pseudorandomized, and the interstimulus interval varied randomly between 10 and 15 seconds (rectangular distribution). An auditory tone delivered between 3 and 6 seconds after the laser pulse (rectangular distribution) prompted the subjects to rate the intensity of pain perception elicited by the laser stimulus, using the 0 to 10 numerical rating scale.

#### 2.2.3. Electroencephalogram recording and data analysis

Participants were seated in a comfortable chair in a silent temperature-controlled room. They wore protective goggles and were asked to focus their attention on the stimuli and relax their muscles. Electroencephalogram data were recorded using 64 Ag-AgCl scalp electrodes placed according to the international 10-20 system (Brain Products GmbH; pass band: 0.01-100 Hz; sampling rate: 1000 Hz). The nose was used as reference, and electrode impedances were kept <10 kΩ. Electro-oculographic signals were simultaneously recorded using surface electrodes, to monitor ocular movements and eye blinks.

Electroencephalogram data were processed using EEGLAB.^8^ Continuous data were band-pass filtered between 1 and 30 Hz. Epochs were extracted using a window analysis time of 3000 milliseconds (from 1000 milliseconds before stimulus to 2000 milliseconds after stimulus) and baseline corrected using the prestimulus interval. Trials contaminated by eye-blinks and movements were corrected using an independent component analysis algorithm.^8^

For each subject and experimental condition, all epochs were averaged, time-locked to the stimulus onset, across all stimulus energies. This procedure yielded 4 average waveforms for each subject, one for each condition (hand and foot, with and without noise). Peak latencies and amplitudes of the Aδ-N2, Aδ-P2, C-N2, and C-P2 waves were measured from the average waveform at Cz, for each subject and experimental condition.^17^

### 2.3. Statistical analyses

For both animal and human experiments, data are expressed as mean ± SD. Statistical analyses consisted of a 2-way repeated-measures analysis of variance (Experiments 1, 2, 4, 5, and 6) and a paired-sample *t* test (Experiments 2 and 4). Statistical comparisons were performed using SPSS (version 20). The level of statistical significance was set at 0.05.

## 3. Results

**Figure 1:**
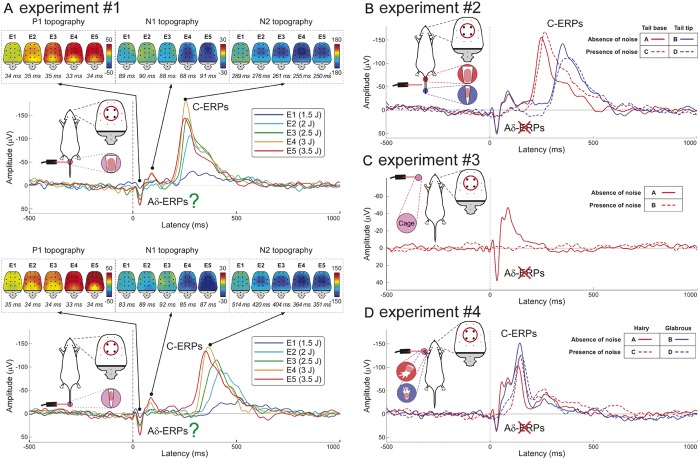
The early part of laser-evoked brain responses reflects the activation of the rat auditory system. (A) Latency of the early “Aδ-ERPs” (P1 and N1 waves) is not dependent on stimulation site (tail base vs tail tip), whereas latency of the late “C-ERPs” (N2 wave) is shorter after proximal than distal stimulations. (B) “Aδ-ERPs” disappear during ongoing noise, whereas “C-ERPs” are unaffected. (C) When laser stimuli are not delivered on the skin, but on the cage surrounding the animal, they still elicit the “Aδ-ERPs,” which, still, disappear during ongoing noise. (D) Both “Aδ-ERPs” and “C-ERPs” are identical when the stimuli are delivered on hairy and glabrous skin. Red circles highlight electrodes (ie, FL2, FR2, PL1, and PR1) from which the plotted ERP waveforms were measured. All waveforms represent group averages. ERPs, event-related potentials.

In Experiment 1, we characterized the stimulus–response function of rat laser-ERPs. They consisted of 2 transient responses, traditionally ascribed to the central arrival of the Aδ- and C-nociceptor afferent volleys^21,46,52^ (Fig. 1A; means and statistics are summarized in Supplementary Tables 1 and 2, available online as Supplemental Digital Content at http://links.lww.com/PAIN/A143). Amplitudes of “Aδ-ERPs” and “C-ERPs” were positively related to stimulus energy. Although “C-ERP” latency was clearly modulated by tail stimulation site (F = 438.07 and *P* < 0.001), “Aδ-ERP” latency was independent of stimulation site (F = 0.33 and *P* = 0.58). The lack of effect of stimulation site on “Aδ-ERP” latency was surprising, as the proximal stimulation (eg, the base of the tail) should yield shorter-latency ERPs than distal stimulation (eg, the tip of the tail). It could have been caused either by volume conduction effect resulting in the difficulty of distinguishing neural activities with different latencies^44^ or by the fact that the “Aδ-ERP” does not reflect the activation of the somatosensory system. This reasoning prompted us to further investigate the functional significance of the “Aδ-ERPs,” in the following 3 experiments. Note that, when examining the effect of stimulation site and stimulus energy on the “C-ERP” latency, there was a significant interaction (F = 9.91 and *P* < 0.01). This interaction indicates that the latency variability of C-ERPs is highly dependent on the length of the C afferents activated by the stimulus: the longer the transmitting pathway, the larger the latency variability of cortical responses.

In Experiment 2, we delivered laser pulses at different sites (tail base and tail tip), with and without ongoing auditory white noise. Early “Aδ-ERPs” were abolished by ongoing noise, whereas late “C-ERPs” were unaffected (Fig. 1B; means and statistics are reported in Supplementary Tables 3–5, available online as Supplemental Digital Content at http://links.lww.com/PAIN/A143). This result suggests that the early response, previously ascribed to the activation of Aδ-nociceptors,^21,46,52^ could rather reflect the activation of the auditory system. This is a viable explanation, given that when laser stimuli are delivered on the skin, they not only induce the temperature increase that activates nociceptors,^54^ but also generate a broadband ultrasound through a thermo-elastic mechanism, ie, the sudden thermal expansion due to the heating of the surface of the irradiated material.^31,49^ This is a well-known phenomenon, and high-power solid-state lasers (like Nd:YAG and CO_2_ lasers used in pain research) are typically used to generate ultrasounds in industrial applications.

The “C-ERP” latency, both with and without ongoing noise, was shorter at proximal than distal stimulation sites (Fig. 1B). The conduction velocity of peripheral afferents mediating the “C-ERPs” was 0.79 ± 0.19 m/s, a range reflecting the physiological properties of C-fibres.^19^ These findings confirmed that “C-ERPs” truly reflected the activation of the somatosensory system.

In Experiment 3, we did not stimulate the skin but delivered the laser stimuli on the cage, at a distance of ∼5 to 10 cm from the animal (Fig. 1C; Supplementary Table 6, available online as Supplemental Digital Content at http://links.lww.com/PAIN/A143). Even without somatosensory stimulation, laser pulses elicited a response virtually identical to the “Aδ-ERPs” observed after skin stimulation in Experiments 1 to 2. Crucially, such “Aδ-ERPs” disappeared when the laser stimulation was delivered on the cage during ongoing noise. These observations provided additional evidence that “Aδ-ERPs” reflected the activation of the auditory system.

Why there is no electrocortical evidence of Aδ-nociceptor activation in rats? Given that some human studies suggest that Aδ-nociceptors are difficult to be activated in glabrous skin,^56^ in Experiment 4, we stimulated both hairy and glabrous skin, with and without ongoing white noise. Without ongoing noise, laser pulses elicited a clear “Aδ-ERP” and “C-ERP” in both hairy and glabrous skin. In contrast, ongoing noise selectively abolished the early “Aδ-ERP,” in both skin types, whereas the late “C-ERP” was unaffected (Fig. 1D, Supplementary Fig. 2; Supplementary Tables 7–9, available online as Supplemental Digital Content at http://links.lww.com/PAIN/A143). Because ERPs truly reflecting Aδ-nociceptor activation are commonly recorded in humans,^17^ the observation that an equivalent response cannot be recorded in rats might seem surprising. However, also in the nociceptive system, there are wide variations of sensitivity across species, and Aδ-fibres are much less easily activated by heat in rats than humans.^9,33^

**Figure 2:**
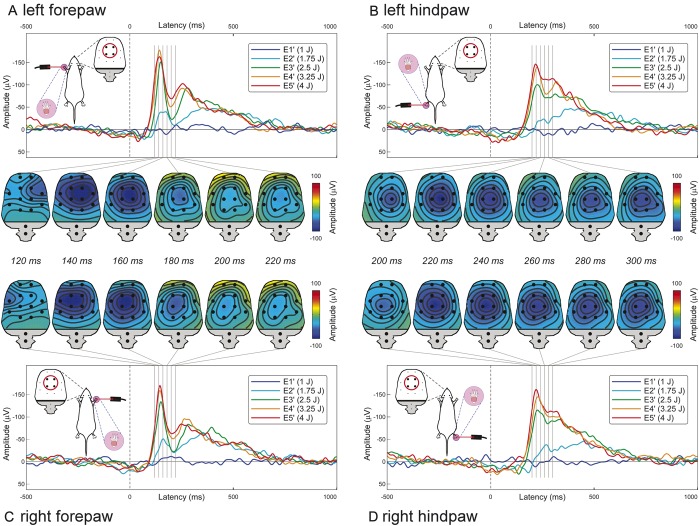
“C-ERP” waveforms and topographies at different stimulation sites and intensities during ongoing noise. The early part of forepaw “C-ERPs” shows a maximum contralateral to the stimulated side (A and C), whereas the early part of hindpaw “C-ERPs” is more centrally distributed (B and D). This topographical difference reflects a source in the primary somatosensory cortex. Red circles highlight electrodes (ie, FL2, FR2, PL1, and PR1) from which the plotted ERP waveforms were measured. All waveforms represent group averages. ERPs, event-related potentials.

**Figure 3:**
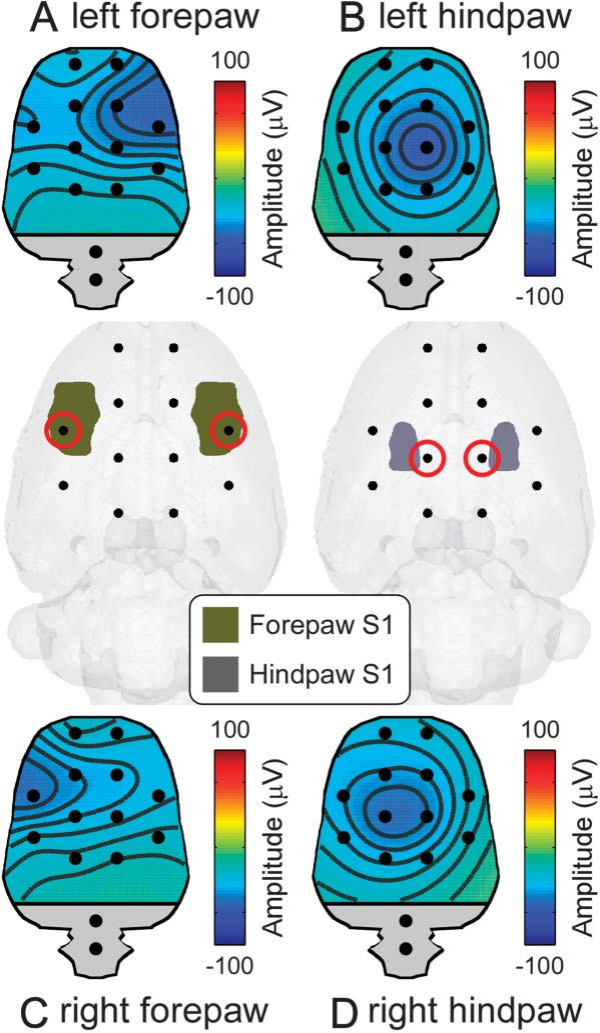
Correspondence between spatial locations of the early part of C-ERPs and the anatomical S1 locations. The early part of the C-ERPs elicited by forepaw (120 milliseconds, A and C) and hindpaw stimulation (200 milliseconds, B and D) showed a negative maximum on the electrodes (red circles) overlying the S1 representation of the stimulated body part. ERPs, event-related potentials.

In Experiment 5, we characterized the physiological properties of “C-ERPs,” after avoiding the early auditory response by delivering ongoing noise (Fig. 2). The early part of the “C-ERPs” elicited by forepaw stimulation (∼120 milliseconds) was maximal contralaterally to the stimulated side, whereas that of the “C-ERPs” elicited by hindpaw stimulation (∼200 milliseconds) was centrally distributed. This topographical difference reflects the somatotopical organization of the primary somatosensory cortex (Fig. 3).^21^ Similar to “C-ERP” amplitudes, scores of nocifensive behaviors were positively related to stimulus energy (F = 161.2 and *P* < 0.001; means and statistics are reported in Supplementary Tables 10 and 11, available online as Supplemental Digital Content at http://links.lww.com/PAIN/A143).

**Figure 4:**
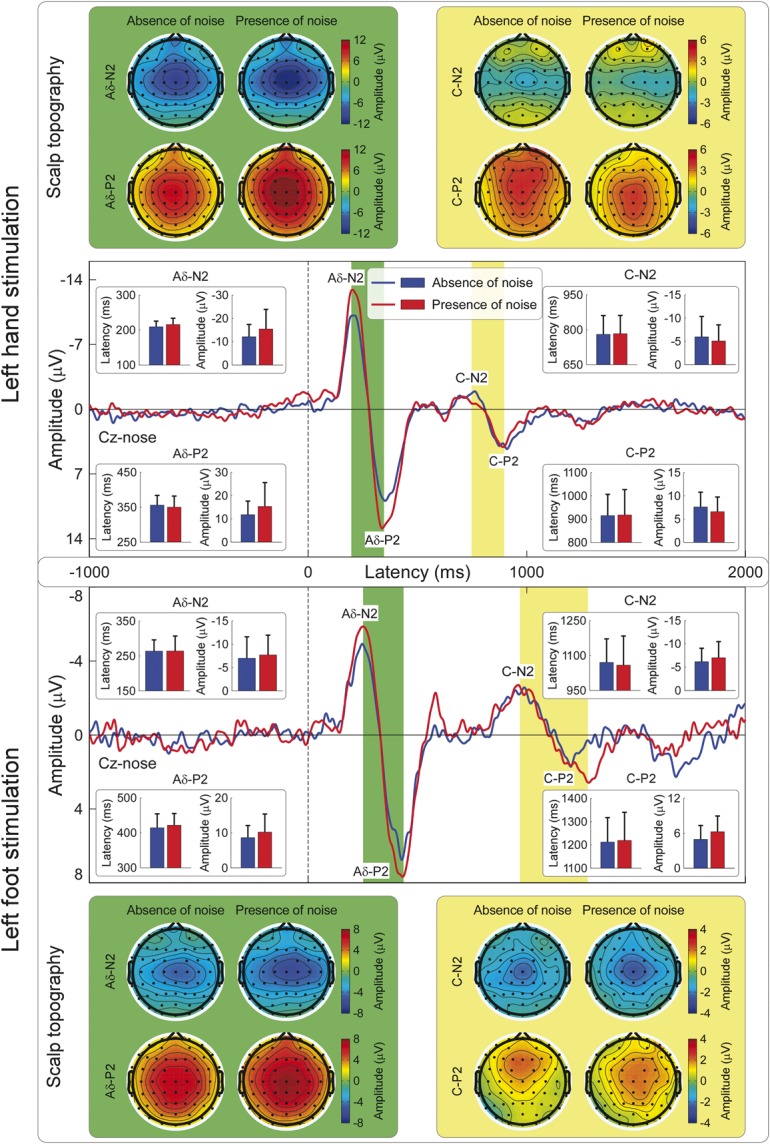
“Aδ-ERPs” and “C-ERPs” in humans: effect of stimulation site and ongoing noise. Both “Aδ-ERPs” and “C-ERPs” have shorter peak latencies when laser stimuli are delivered to the hand (upper panel) compared with the foot (lower panel) (*P* < 0.001 for all comparisons: Aδ-N2, Aδ-P2, C-N2, and C-P2; Supplementary Table 13, available online as Supplemental Digital Content at http://links.lww.com/PAIN/A143). The conduction velocity based on the latency difference of the early “Aδ-ERPs” (13.5 ± 8.8 m/s) was compatible with the conduction velocity of Aδ afferents. The conduction velocity based on the latency difference of the late “C-ERPs” (1.9 ± 0.3 m/s) was compatible with the conduction velocity of C afferents. Importantly, latencies and amplitudes of both “Aδ-ERPs” and “C-ERPs” were minimally affected by ongoing noise (*P* > 0.05 for all comparisons expect Aδ-N2 amplitude; Supplementary Table 13, available online as Supplemental Digital Content at http://links.lww.com/PAIN/A143). All waveforms represent group averages. ERPs, event-related potentials.

In Experiment 6, we characterized the physiological properties of laser-evoked ERPs in humans, and tested whether they are affected by the presence of ongoing white noise during the recording session. This experiment yielded 2 main results. First, as previously observed in a different sample of 34 healthy participants,^17^ we confirmed that the laser-evoked ERPs consisted of 2 transient responses: a first response occurring in a time window compatible with the conduction velocity of Aδ afferents (“Aδ-ERPs”), followed by a response occurring in a time window compatible with the conduction velocity of C afferents (“C-ERPs”) (Fig. 4). The conduction velocities of the afferent pathways mediating the “Aδ-ERPs” and “C-ERPs” were 13.5 ± 8.8 m/s and 1.9 ± 0.3 m/s, respectively. These values were estimated in each individual, by dividing the difference in the latencies of the responses elicited by hand and foot stimulation by the difference in their conduction distances.^19^ Second, and more important for the issue addressed in this study, both early “Aδ-ERP” and late “C-ERP” latencies and amplitudes were minimally affected by ongoing noise (Fig. 4; Supplementary Tables 12 and 13, available online as Supplemental Digital Content at http://links.lww.com/PAIN/A143). This result is in striking contrast with what observed in rat laser-ERPs. Altogether, these findings confirmed that “Aδ-ERPs” and “C-ERPs” truly reflected the activation of the somatosensory system in humans. Note that, as observed in previous human studies,^17^ the “C-ERP” amplitude was smaller than the amplitude of the preceding “Aδ-ERPs” (Fig. 4). This amplitude difference is not surprising, given that (1) the saliency of the eliciting stimulus is a major determinant of laser-evoked ERPs^18,40,47^ and (2) the saliency of the Aδ-fiber input is higher than that of the following C-fibre input, because of the lower intensity and higher temporal predictability of the latter.^17^ Keeping this in mind, the observation that the early auditory response recorded in rats had a smaller amplitude than the late “C-ERPs” indicates that the early auditory input is probably less intense than the subsequent somatosensory input.

## 4. Discussion

We demonstrated that the early component of the laser-evoked rat electrocortical response, widely interpreted as reflecting the activation of Aδ-nociceptors,^21,28,46,51–53,57,62^ results instead from the activation of the auditory system by the ultrasounds generated by laser stimuli through a thermo-elastic mechanism. This laser-evoked auditory response is not detected in humans, whose auditory system is insensitive to ultrasounds. In contrast, the late component of the laser-evoked electrocortical response truly reflects the activation of the rat nociceptive system.

That the so-called “Aδ-ERPs” in rats reflect the activation of the auditory system is supported by 4 important pieces of evidence. First, the “Aδ-ERP” latency was independent of stimulation site (Fig. 1A). This observation is in contrast with the notion that the stimulation of proximal and distal sites (eg, tail base and tail tip, respectively) elicit cortical responses at significantly different latencies when the somatosensory volleys are transmitted through peripheral Aδ afferents with a relatively slow conduction velocity of ∼15 m/s.^51^ Second, early “Aδ-ERPs” were completely abolished by ongoing white noise (Fig. 1B–D), an observation in contrast with several human studies reporting clear Aδ-related brain responses recorded using an equivalent amount of ongoing white noise (Fig. 4).^3^ Third, laser pulses delivered on the cage at a distance of ∼5 to 10 cm from the animal elicited a brain response similar to the “Aδ-ERPs” (Fig. 1C). Fourth, in both hairy and glabrous skin, laser pulses elicited identical “Aδ-ERPs,” which were both abolished by ongoing white noise (Fig. 1D). This finding ruled out the possibility that the absence of “Aδ-ERPs” in rats was due to the difficulty of activating Aδ nociceptors in glabrous skin, as previously suggested.^56^ Importantly, our conclusion that “Aδ-ERPs” reflect the activation of the rat auditory system is not contradicted by the observation that the “Aδ-ERP” amplitude was positively related to energy of the laser stimulus (Fig. 1A). Indeed, a laser pulse with more energy generates a stronger ultrasound because of a more effective thermo-elastic effect.^49^

Taken together, these observations indicate that the widely accepted notion that the early component of laser-evoked ERPs in rats reflects the activation of Aδ nociceptors (eg, Refs. 21,^28^,^46^,^51^,^52^,^53^,^57^,^62^) is a misconception. It is important to highlight that the explanation that “Aδ-ERPs” reflect the activation of the auditory system can be also put forward when interpreting the laser-evoked neural responses recorded invasively using microelectrodes (eg, local field potentials and spikes) and even when the microelectrodes are located in the primary somatosensory cortex.^28,53,60^ Indeed, primary sensory cortices, traditionally regarded as unisensory, have been shown to also respond to isolated sensory stimuli of a range of different sensory modalities.^29,32^ An important implication of the present results is that future studies using laser stimuli to investigate the neural mechanisms of nociception and pain should take care of excluding the contribution of the auditory system to the recorded responses. This is relevant given the growing number of studies using laser nociceptive stimulation in rat assays of nociception and pain.^5,11,22,26,35,36,50,59^

Another implication of these results is that “C-ERPs” in rats truly reflect the activation of unmyelinated somatosensory pathways. This is supported by the following arguments. First, the “C-ERP” latency was clearly modulated by stimulation site along the tail (Fig. 1A), with significantly shorter-latency responses elicited by the stimulation of more proximal sites. Considering the distance between stimulation sites, the latency difference indicated a conduction velocity of 0.79 ± 0.19 m/s (Fig. 1B), a range reflecting the physiological properties of C afferent fibres.^19,27^ Second, the “C-ERP” amplitude was modulated by stimulus energy, with responses of larger amplitude at stronger stimulus energies (Fig. 1A). Third, “C-ERPs” were unaffected by ongoing white noise (Fig. 1B–D), thus ruling out the possible contribution of the activation of the auditory system. Finally, the spatial distribution of the early part of the “C-ERPs” elicited by forepaw stimulation showed a maximum contralateral to the stimulated side, whereas that of the early part of the “C-ERPs” elicited by hindpaw stimulation was more centrally distributed (Fig. 2). This topographical difference strictly follows the somatotopical organization of the primary somatosensory cortex (Fig. 3).

It should be noted that additional works needs to be done to determine whether the brain responses elicited using different laser devices (eg, solid-state Nd:YAP and CO2 lasers, which have different wavelengths and pulse duration^45^) are all confounded by the activation of the auditory system. Also, given the increasing use of mice in pain research, it will be important to test whether these results generalize to other murine models of nociception.

In summary, our study provides compelling evidence that laser-generated ultrasounds are detected by the rat auditory system, and evoke a response that has been so far mistakenly interpreted as reflecting the Aδ-somatosensory input in several studies (eg,Refs. 21,^28^,^46^,^51^,^52^,^53^,^57^,^62^). The observation that rats respond first to the acoustic stimulation generated by the laser, before the afferent nociceptive volley reaches the brain, highlights an important limitation of laser stimulation as currently used in rat studies. This conclusion has wide implications, not only retrospectively, as it prompts a reconsideration of previous interpretations based on the so-called “Aδ-ERPs” in basic, preclinical, and pharmacological research,^46,52,57^ but also prospectively, as laser stimulation is considered one of the best tools to investigate pain psychophysics in rats and humans, and is therefore being increasingly used.^5,7,11–13,22,24–26,28,35,36,45,46,48,50,52,55,57,59^ These results have implications in the interpretation of results obtained with other nociceptive assays. For example, the Hargreaves test, which also uses radiant heat, is likely to mostly reflect the function of C-fibres. Translational studies in animals are key to furthering our understanding of clinical conditions and bridging the gaps between new molecules and patients.^37,38,58^ The validity of such translations critically relies on a careful consideration of the different physiological properties of sensory systems across species.

## Conflict of interest statement

The authors have no conflicts of interest to declare.

L. Hu is supported by the National Natural Science Foundation of China (31200856 and 31471082) and Key Laboratory of Mental Health, Institute of Psychology, Chinese Academy of Sciences. G.D. Iannetti acknowledges the support of the Royal Society and the Wellcome Trust. The collaboration between L. Hu and G.D. Iannetti is supported by the IASP Developed-Developing Countries Collaborative Research Grant. The funders had no role in study design, data collection and analysis, decision to publish, or preparation of the manuscript.

## Supplementary Material

SUPPLEMENTARY MATERIAL

## Appendix A Supplemental Digital Content

Supplemental Digital Content associated with this article can be found online at http://links.lww.com/PAIN/A143.
