# Supplementary material for: Was it a pain or a sound? Across-species variability in sensory sensitivity
Source: Pain. 2015 Aug 6;156(12):2449–57. doi: 10.1097/j.pain.0000000000000316 (PMC4764020; doi:10.1097/j.pain.0000000000000316)
Supplement: SUPPLEMENTARY MATERIAL [file jop-156-2449-s001.docx]

**
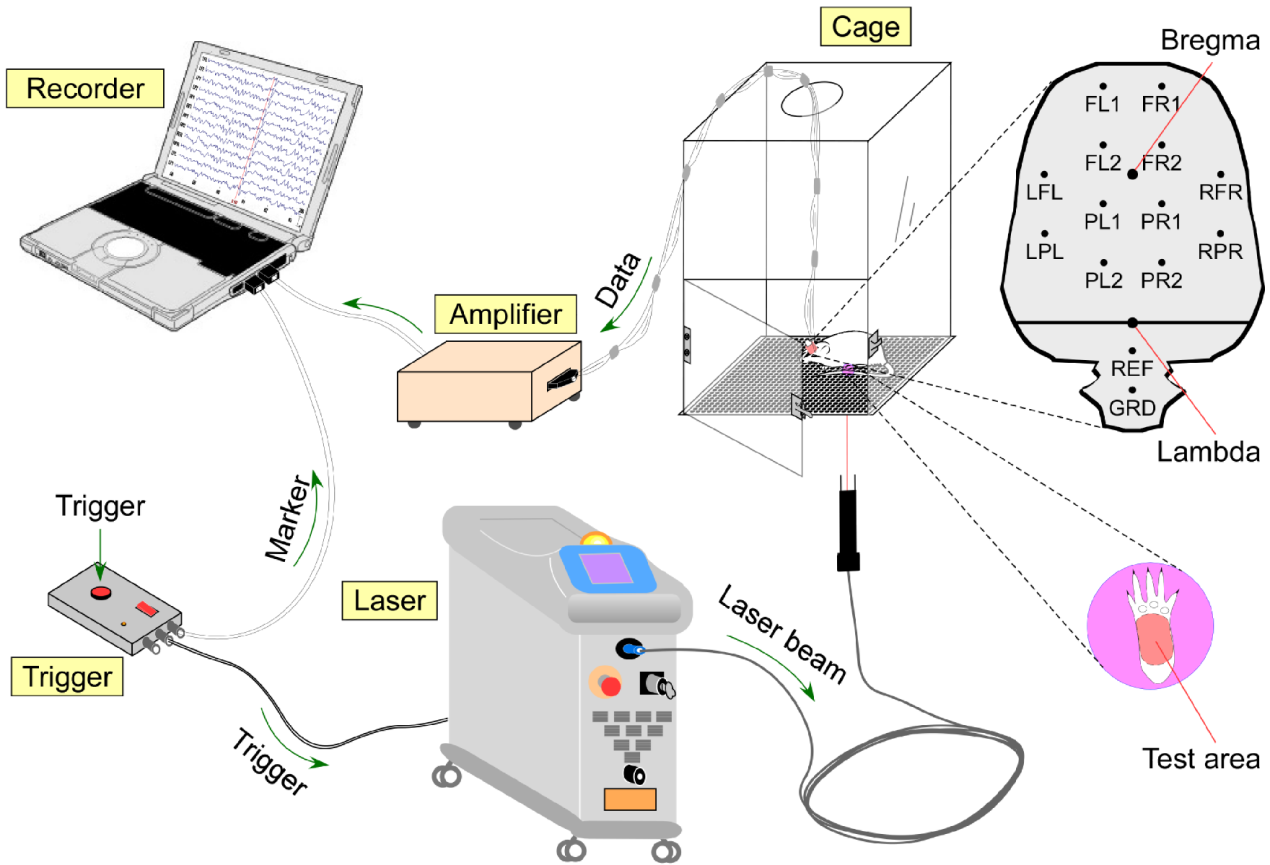
**

**Supplementary Fig. 1. Schematic of the setup to record laser-evoked ERPs in rats.**

Infrared neodymium yttrium aluminum perovskite (Nd:YAP) laser pulses activate selectively nociceptive terminals in the superficial skin layers. Laser pulses are delivered through an optic fibre to the freely moving rat. EEG signals are transmitted through flexible wires to the EEG amplifier and recording system. EEG data are recorded from 14 electrodes placed according to stereotaxic coordinates. The coordinates of 12 active electrodes are as follows (positions are expressed in respect to the Bregma, in mm; positive X and Y axis values indicate right and anterior locations, respectively). FL1: X=-1.5, Y=4.5; FR1: X=1.5, Y=4.5; FL2: X=-1.5, Y=1.5; FR2: X=1.5, Y=1.5; LFL: X=-4.5, Y=0; RFR: X=4.5, Y=0; PL1: X=-1.5, Y=-1.5; PR1: X=1.5, Y=-1.5; LPL: X=-4.5, Y=-3; RPR: X=4.5, Y=-3; PL2: X=-1.5, Y=-4.5; PR2: X=1.5, Y=-4.5. Reference (REF) and ground (GRD) electrodes are respectively placed at 2 mm and 4 mm caudally to the Lambda, on the midline.

**
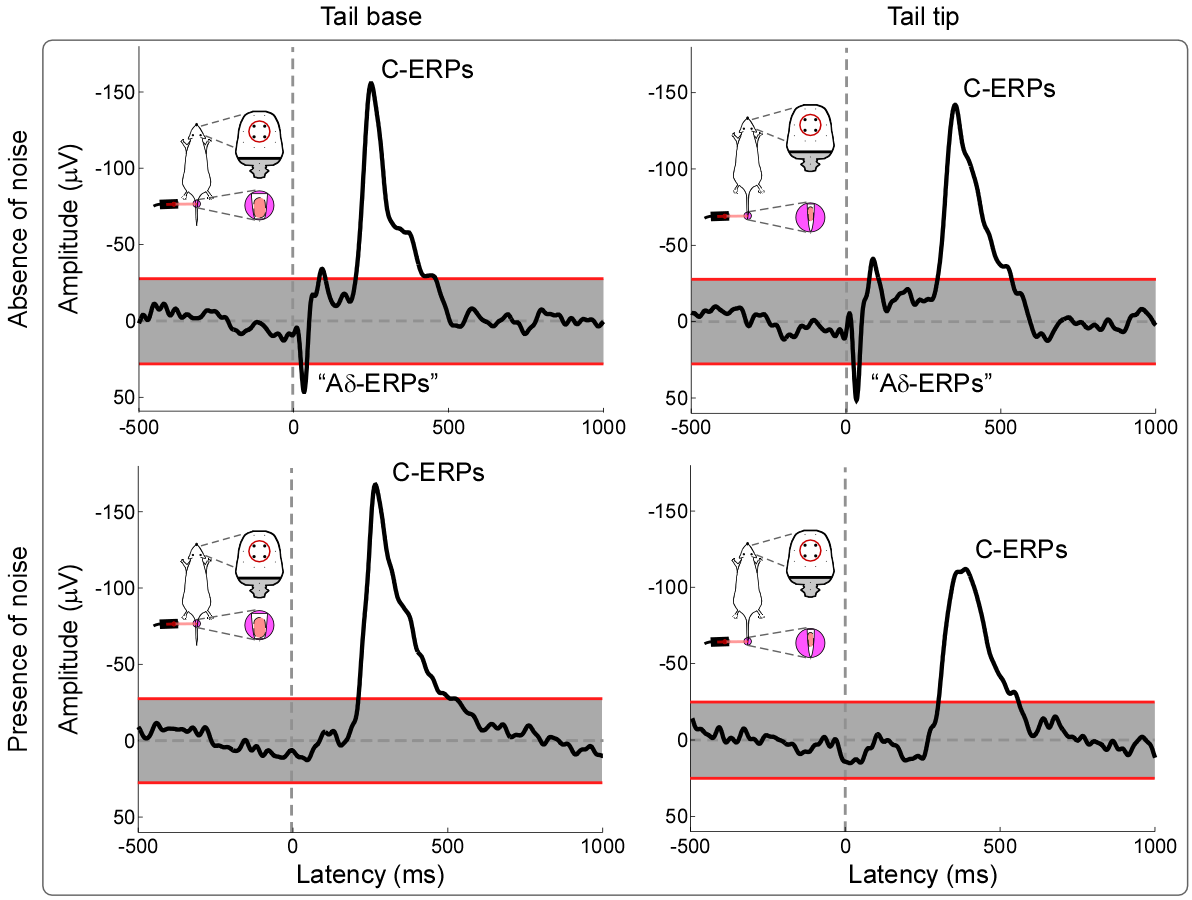
**

**Supplementary Fig. 2. Statistical assessment of the significance of the laser-evoked brain responses (Experiment 2).**

Without ongoing white noise, both early “Aδ-ERP” and late “C-ERP” responses were significantly different from the pre-stimulus variability (top panels). With ongoing white noise, only the late “C-ERP” responses were significantly different from the pre-stimulus variability, whereas the early “Aδ-ERP” responses were not (bottom panels). The red lines indicate the p=0.05 threshold (corresponding to 1.96 times the averaged variability – estimated as SD – of the pre-stimulus fluctuations across subjects).

**Supplementary Table 1**. Latency and amplitude of ERPs elicited by laser stimulation at different sites (tail base and tip) and energies (E1-E5) (Experiment 1).

|  | Tail base | | | | |  | Tail tip | | | | |
| --- | --- | --- | --- | --- | --- | --- | --- | --- | --- | --- | --- |
|  | E1 (1.5 J) | E2 (2 J) | E3 (2.5 J) | E4 (3 J) | E5 (3.5 J) |  | E1 (1.5 J) | E2 (2 J) | E3 (2.5 J) | E4 (3 J) | E5 (3.5 J) |
| P1 latency (ms) | 33±5 | 36±6 | 37±6 | 34±4 | 35±6 |  | 35±9 | 35±6 | 35±4 | 34±4 | 35±5 |
| P1 amplitude (μV) | 12.5±12.6 | 29.5±20.1 | 38.3±27.5 | 48.8±31.7 | 53.2±30.5 |  | 10.3±15.8 | 28.9±20.5 | 34.8±29.7 | 49.6±30.3 | 51.2±32.1 |
| N1 latency (ms) | 90±10 | 87±7 | 90±12 | 89±9 | 88±6 |  | 89±11 | 86±9 | 89±11 | 88±7 | 90±7 |
| N1 amplitude (μV) | -9.3±9.4 | -4.8±18.4 | -17.0±22.2 | -20.7±36.8 | -29.6±33.5 |  | -9.0±7.8 | -2.9±18.1 | -17.3±21.6 | -14.3±34.2 | -30.3±36.0 |
| N2 latency (ms) | 284±39 | 290±27 | 272±22 | 257±10 | 255±16 |  | 507±35 | 423±28 | 398±31 | 367±18 | 353±32 |
| N2 amplitude (μV) | -48.9±29.2 | -122.7±61.7 | -159.1±74.5 | -186.7±60 | -167.8±42.7 |  | -37.6±22.9 | -113.5±58.7 | -137.4±60.3 | -152.5±66.0 | -147.6±54.8 |

**Supplementary Table 2**. Two-way repeated-measures analysis of variance (ANOVA) exploring the effect of stimulation site (tail base vs. tip) and stimulus energy (E1-E5) on laser ERPs (Experiment 1).

|  | Stimulation site | |  | Stimulus energy | |  | Interaction | |
| --- | --- | --- | --- | --- | --- | --- | --- | --- |
|  | F value | p value |  | F value | p value |  | F value | p value |
| P1 latency | 0.02 | 0.89 |  | 0.68 | 0.61 |  | 1.13 | 0.41 |
| P1 amplitude | 1.48 | 0.25 |  | 11.56 | <0.001*** |  | 0.83 | 0.55 |
| N1 latency | 0.33 | 0.58 |  | 0.24 | 0.92 |  | 0.53 | 0.72 |
| N1 amplitude | 0.93 | 0.36 |  | 2.83 | <0.05* |  | 0.59 | 0.68 |
| N2 latency | 438.07 | <0.001*** |  | 66.58 | <0.001*** |  | 9.91 | <0.01** |
| N2 amplitude | 7.64 | <0.05* |  | 19.87 | <0.001*** |  | 0.99 | 0.43 |

*p<0.05; **p<0.01; ***p<0.001.

**Supplementary Table 3**. Latency and amplitude of ERPs elicited by laser stimulation at different tail sites (base and tip) and noise conditions (presence and absence of ongoing white noise) (Experiment 2).

|  | Tail base | | Tail tip | | |
| --- | --- | --- | --- | --- | --- |
|  | Absence of noise | Presence of noise |  | Absence of noise | Presence of noise |
| P1 latency (ms) | 34±7 | — |  | 32±8 | — |
| P1 amplitude (μV) | 52.6±30.2 | — |  | 54.2±36.7 | — |
| N1 latency (ms) | 90±8 | — |  | 88±6 | — |
| N1 amplitude (μV) | -26.8±38.7 | — |  | -34.3±39.9 | — |
| N2 latency (ms) | 254±15 | 262±19 |  | 352±33 | 366±28 |
| N2 amplitude (μV) | -175.0±42.0 | -194.1±78.3 |  | -155.7±53.0 | -156.4±66.9 |

**Supplementary Table 4**. Paired sample t-test exploring the effect of stimulation site (tail base vs. tip) on “Aδ-ERPs” (Experiment 2).

| Paired sample t-test (stimulation site) | | |
| --- | --- | --- |
|  | t value | p value |
| P1 latency | 1.21 | 0.25 |
| P1 amplitude | 0.24 | 0.81 |
| N1 latency | 1.21 | 0.25 |
| N1 amplitude | 0.86 | 0.41 |

**Supplementary Table 5**. Two-way repeated-measures ANOVA exploring the effect of stimulation site (tail base vs. tip) and noise condition (presence vs. absence of ongoing white noise) on “C-ERPs” (Experiment 2).

| Two way repeated-measures ANOVA | | | | | | | | |
| --- | --- | --- | --- | --- | --- | --- | --- | --- |
|  | Stimulation site | |  | Noise condition | |  | Interaction | |
|  | F value | p value |  | F value | p value |  | F value | p value |
| N2 latency | 361.70 | <0.001*** |  | 3.87 | 0.08 |  | 0.35 | 0.57 |
| N2 amplitude | 9.38 | <0.05* |  | 0.43 | 0.53 |  | 1.93 | 0.19 |

*p<0.05; ***p<0.001.

**Supplementary Table 6**. Latency and amplitude of “Aδ-ERPs” elicited when laser stimuli are not delivered on the skin, at different noise conditions (presence and absence of ongoing white noise) (Experiment 3).

|  | Absence of noise | Presence of noise |
| --- | --- | --- |
| P1 latency (ms) | 34±3 | — |
| P1 amplitude (μV) | 39.6±27.5 | — |
| N1 latency (ms) | 86±3 | — |
| N1 amplitude (μV) | -48.1±45.0 | — |

**Supplementary Table 7**. Latency and amplitude of ERPs elicited by laser stimulation of hairy and glabrous skin, at different noise conditions (presence and absence of ongoing white noise) (Experiment 4).

|  | Hairy | | Glabrous | | |
| --- | --- | --- | --- | --- | --- |
|  | Absence of noise | Presence of noise |  | Absence of noise | Presence of noise |
| P1 latency (ms) | 30±4 | — |  | 31±3 | — |
| P1 amplitude (μV) | 42.6±35.0 | — |  | 37.2±25.3 | — |
| N1 latency (ms) | 84±5 | — |  | 84±7 | — |
| N1 amplitude (μV) | -77.1±58.5 | — |  | -68.8±39.8 | — |
| N2 latency (ms) | 137±13 | 143±14 |  | 145±9 | 147±12 |
| N2 amplitude (μV) | -139.0±43.0 | -146.4±64.3 |  | -165.2±52.8 | -151.4±67.9 |

**Supplementary Table 8**. Paired sample t-test exploring the effect of skin type (hair vs. glabrous) on “Aδ-ERPs” (Experiment 4).

|  | t value | p value |
| --- | --- | --- |
| P1 latency | 0.80 | 0.44 |
| P1 amplitude | 0.70 | 0.50 |
| N1 latency | 0.45 | 0.67 |
| N1 amplitude | 0.57 | 0.58 |

**Supplementary Table 9**. Two-way repeated-measures ANOVA exploring the effect of skin type (hair vs. glabrous) and noise condition (presence vs. absence of white noise) on “C-ERPs” (Experiment 4).

| Two way repeated-measures ANOVA | | | | | | | | |
| --- | --- | --- | --- | --- | --- | --- | --- | --- |
|  | Stimulation site | |  | Noise condition | |  | Interaction | |
|  | F value | p value |  | F value | p value |  | F value | p value |
| N2 latency | 3.62 | 0.08 |  | 13.38 | <0.01** |  | 0.81 | 0.39 |
| N2 amplitude | 1.96 | 0.19 |  | 0.03 | 0.86 |  | 2.97 | 0.11 |

**p<0.01.

**Supplementary Table 10**. Behavioral scores at different stimulus intensities (E1'-E5') and stimulation sites (left forepaw, right forepaw, left hindpaw, and right hindpaw) (Experiment 5).

|  | E1' (1 J) | E2' (1.75 J) | E3' (2.5 J) | E4' (3.25 J) | E5' (4 J) |
| --- | --- | --- | --- | --- | --- |
| Left forepaw | 0.03±0.06 | 1.83±0.93 | 3.09±0.25 | 3.29±0.15 | 3.51±0.11 |
| Right forepaw | 0.09±0.15 | 2.01±0.82 | 2.96±0.35 | 3.37±0.15 | 3.52±0.18 |
| Left hindpaw | 0.04±0.08 | 1.65±0.98 | 2.91±0.48 | 3.35±0.17 | 3.53±0.17 |
| Right hindpaw | 0.01±0.04 | 1.58±0.85 | 2.89±0.50 | 3.37±0.23 | 3.59±0.18 |

**Supplementary Table 11**. Two-way repeated-measures ANOVA exploring the effect of stimulation site (left forepaw, right forepaw, left hindpaw, right hindpaw) and stimulus energy (E1'-E5') on behavioural scores obtained after laser stimulation (Experiment 5).

| Two way repeated-measures ANOVA | | | | | | | | |
| --- | --- | --- | --- | --- | --- | --- | --- | --- |
|  | Stimulation site | |  | Stimulus energy | |  | Interaction | |
|  | F value | p value |  | F value | p value |  | F value | p value |
| Behavioural score | 1.94 | 0.14 |  | 161.20 | <0.001*** |  | 2.56 | 0.06 |

***p<0.001.

**Supplementary Table 12**. Latency and amplitude of ERPs elicited by laser stimulation at different stimulation sites (left hand and foot) and noise conditions (presence and absence of ongoing white noise) (Experiment 6).

|  | Left hand | | Left foot | | |
| --- | --- | --- | --- | --- | --- |
|  | Absence of noise | Presence of noise |  | Absence of noise | Presence of noise |
| Aδ-N2 latency (ms) | 209±18 | 216±19 |  | 264±33 | 264±44 |
| Aδ-N2 amplitude (μV) | -12.1±5.4 | -15.4±8.6 |  | -6.98±4.60 | -7.68±4.26 |
| Aδ-P2 latency (ms) | 356±29 | 350±33 |  | 415±41 | 422±33 |
| Aδ-P2 amplitude (μV) | 11.8±6.0 | 15.3±10.4 |  | 8.63±3.61 | 10.20±5.32 |
| C-N2 latency (ms) | 780±82 | 783±80 |  | 1070±103 | 1059±127 |
| C-N2 amplitude (μV) | -5.96±4.45 | -5.09±3.53 |  | -6.16±2.96 | -7.01±3.53 |
| C-P2 latency (ms) | 915±93 | 918±110 |  | 1212±107 | 1219±122 |
| C-P2 amplitude (μV) | 7.63±3.24 | 6.59±3.23 |  | 4.94±2.44 | 6.26±2.75 |

**Supplementary Table 13**. Two-way repeated-measures ANOVA exploring the effect of stimulation site (left hand vs. foot) and noise condition (presence vs. absence of ongoing white noise) on “Aδ-ERPs” and “C-ERPs” in humans (Experiment 6).

| Two way repeated-measures ANOVA | | | | | | | | |
| --- | --- | --- | --- | --- | --- | --- | --- | --- |
|  | Stimulation site | |  | Noise condition | |  | Interaction | |
|  | F value | p value |  | F value | p value |  | F value | p value |
| Aδ-N2 latency | 33.25 | <0.001*** |  | 0.30 | 0.60 |  | 0.33 | 0.58 |
| Aδ-N2 amplitude | 16.07 | <0.01** |  | 5.24 | <0.05* |  | 1.63 | 0.22 |
| Aδ-P2 latency | 46.89 | <0.001*** |  | 0.01 | 0.92 |  | 0.57 | 0.46 |
| Aδ-P2 amplitude | 4.44 | 0.05 |  | 4.54 | 0.05 |  | 1.14 | 0.30 |
| C-N2 latency | 251.6 | <0.001*** |  | 0.34 | 0.57 |  | 1.28 | 0.28 |
| C-N2 amplitude | 3.37 | 0.09 |  | 0.00 | 0.97 |  | 3.02 | 0.10 |
| C-P2 latency | 359.4 | <0.001*** |  | 0.31 | 0.59 |  | 0.03 | 0.87 |
| C-P2 amplitude | 6.87 | <0.05* |  | 0.10 | 0.76 |  | 9.66 | <0.01** |

*p<0.05; **p<0.01; ***p<0.001.
